# A novel proton-exchange porous silicon membrane production method for μDMFCs

**DOI:** 10.3906/kim-2002-32

**Published:** 2020-08-18

**Authors:** Meltem GÖR BÖLEN, Tevhit KARACALI

**Affiliations:** 1 Department of Electrical and Electronic Engineering, Erzurum Technical University, Erzurum Turkey; 2 Department of Electrical and Electronic Engineering, Atatürk University, Erzurum Turkey

**Keywords:** Porous silicon, μDMFCs, membrane

## Abstract

This study introduces a new production method to use as a porous silicon-based proton exchange membrane for μDMFCs. In this respect, EIS, fuel crossover test, and fuel cell performance test at the μDMFC sample cell are performed at room temperature on a porous silicon-based membrane that was produced for passive mode μDMFC as a proton exchange membrane. The reason for performing the fuel crossover test is to ensure the silicon opened pores along the silicon wafer and to examine the fuel permeability of the membrane. The fuel crossover test shows that the fuel cell provides energy for about 60 min with a 50 mL fuel. EIS reveals proton permeability of proton exchange membrane. The calculated value of the conductivity of the membrane is 0.0016 S/cm. OCV of the system is 0.4V, whereas values (with highest power density is 0.1 mW/cm²and with the highest current density is 0.39 mA/cm²) are low. However, porous silicon is not a natural proton conductor. Hence, these values can be increased by different ways such as porous silicon functionalized, or serial connection of fuel cells. On the other hand, the value of OCV is consistent with the previous studies. In sum, this study presents a simple, cost-effective, and short time-consuming method for the production of porous silicon as proton-conducting membrane behavior.

## 1. Introduction

In the last century, the total production of energy from fossil fuels has increased rapidly, leaving fossil fuel reserves depleted. As the population and industrialization continue to increase, the global energy demand is expected to increase even more sharply in the next few decades. Therefore, the search for an alternative energy source has begun [1–3]. Hydrogen is one such energy source that has gathered much attention as a preferred energy carrier especially for transportation applications [4]. It can be used in fuel cells to produce electricity, or power and heat [5]. Previous studies revealed that unlike other renewable energy sources, hydrogen-based energy systems come to forefront in the total amount of energy they can store [5].

Fuel cells produce electrical energy by an electrochemical reaction between a fuel, typically hydrogen, and the oxygen in the air. While operating efficiently, they are quiet and reliable [1–3]. Among the fuel cell types, micro direct methanol fuel cell (μDMFC) is highly applicable and preferable for portable applications and microsystems due to several factors such as high energy density, recharging and ease of transport, low temperature operation, low pollution, and simple and safe use. Due to these advantages, μDMFC has the potential to become the leading portable power source of the future [6–12] so that the number of studies on μDMFC have increased. The Nafion membrane is mostly used as a proton-conducting membrane. However, it has the following disadvantages: its shape is greatly affected by the environmental conditions; its resistance is quite dominant and it is not compatible with standard microfabrication techniques. These disadvantages have led to the search for a more suitable proton-conducting membrane for μDMFCs. In this respect, the porous silicon, which has a high specific surface area and good mechanical stability, has attracted attention as a proton conducting membrane over the last few years [9].

Previous studies [13,14], demonstrated a new technique of making miniature fuel cells using silicon substrate with porous silicon membrane filled with Nafion. The production process covered the following steps. Firstly, thin thermal oxidation was applied on the surface of Si wafer. Then, a thin layer of Cr/Au film was sputtered onto the silicon oxide layer. That was followed by photoresist deposition and UV insolation through a photolithography mask and development in adapted solution. The next step was the deoxidation of insulated patterns. It was followed by etching with potassium hydroxide (KOH) in order to avoid dispersions when openin pores in silicon. The last step was to make silicon membranes porous by anodization in an ethanolic–hydrofluoric acid (HF) solution. Additionally, thin reactive ion etching (RIE) was performed on membranes from the backside to make sure that all the pores are opened. Another study [15] reported various performance tests of acid loaded porous silicon membrane based micro fuel cells under various parameters and conditions (molarity, thickness). The fabrication process of acid loaded porous silicon membrane starts with deposition of silicon nitride film by low pressure chemical vapor deposition (LPCVD) and then patterned as mask for KOH etching. Using HF solution, electrochemical etching of silicon substrate was performed to form pores, and RIE on silicon substrate backside was applied to open up pores on the backside. Another study [16] examined the performance of an acid loaded porous silicon membrane for micro-fuel cells by following similar fabrication process in a different order. A thin layer of Cr/Au film was deposited for metal electrical contact. It was followed by Photoresist (PR) application and patterning using photolithography process. Then, deep reactive ion etching was performed to achieve silicon membranes of desired thickness. Finally, anodization was performed in ethanolic HF solution. Following the suggested fabrication process of the study [14], Wang et al. [9] presented a Nafion filled porous silicon membrane as proton exchange membrane (PEM). However, instead of RIE, inductively coupled plasma (ICP) was used to make sure that all the pores are opened. Zhou et al. [12] presented a prototype that μDMFC integrated with passive fuel delivery based on a surface tension driving mechanism, and reported performance of the μDMFC at room temperature and 40% relative humidity. SiO2 /Si3 N4 and Cr/Au were deposited on silicon wafer as the mask layers for KOH etching and anodization. Then, applying a microfabrication technique (double-side lithography and KOH etching), the patterns of both-side structures were formed, and etched. The next step was to form the porous silicon membrane by anodization in HF/ethanol solution. As of last, ICP was employed to etch the residual silicon on the backside of the porous silicon membrane. Following the fabrication process of the previous study [9], another study [17] developed a proton exchange membrane based on sulfo functionalized porous silicon for μDMFC. Besides, a detailed analysis related to the performance of presented sulfo functionalized porous silicon was reported in the study. Previous studies conducted fuel cell performance tests that were conducted under a variety of conditions such as different thicknesses of the membrane, fuel molarity, or various ways of functionalized membranes. Hence, detailed information related to the fabrication process of the porous silicon membrane was given in these studies. However, the main steps of the fabrication process that covers some activities such as KOH etching, the Cr/Au thin film deposition, the photolithography method used for masking, ICP, deep reactive ion etching (DRIE) and RIE methods used to ensure that the pore ends are opened, require a long time and include parameter-dependent methods. In order to eliminate these issues; this study presents a new method of porous silicon membrane production that was made for the use of as a membrane at passive mode direct methanol fuel cells. The proposed fabrication process offers a simple, cost, and time-effective way for the production of porous silicon without the need for special devices.

### 2.1. Fabrication of porous silicon membrane

**Figure 1:**
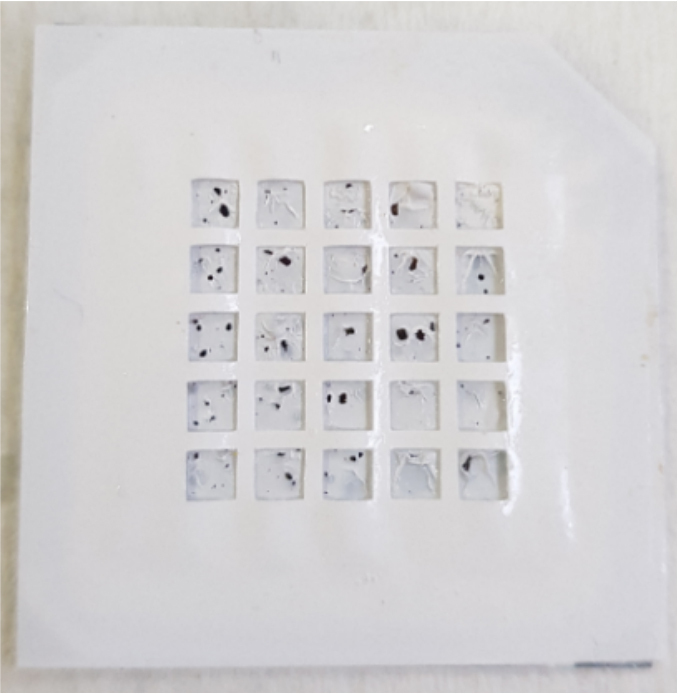
Silicon was masked by Dyo SF-0411 and PVC film.

Our porous silicon membrane was manufactured from 0.001–0.005 Ωcm boron-doped p-type (100) oriented silicon wafer with a thickness of 200 μm. The electrochemically anodic etching was performed using HF solution (1:2 (v/v) HF (40%)/EtOH (99%) ethanol to ensure pore formation in the selected silicon wafer. When the porous silicon has formed, the silicon wafer has masked by using a method that is simple, low cost and completed in a very short time. This new method used for masking consists of 2 steps: Application of Dyo SF-0411 with serigraphy (step 1). Gluing soft PolyVinyl Chloride (PVC) film on the painted surface of the silicon wafer (step 2). After the masking had been completed, an active area of 2.56 cm^2^ was obtained. Anodization was performed to provide pore formation along the 200 μm. Pore formation was preferred by the layered structure where the time and the layer thickness were controlled for the anodization process. For this purpose, 1,2,5, and 10 mA/cm^2^ current densities were applied in a certain order to form a layered structure to provide pore formation. After the anodization process had been completed, –10 V was applied for 25 min. To check that the pores were open, the heated solution was evacuated, and the solution at room temperatur was placed in the cell and reapplied at –10 V for 25 min. Constant current densities and voltage were applied between 2 electrodes with Keithley 2400. After the pore formation, the silicon wafer was purified from HF by deionized water. Figure 1 shows the produced porous silicon.

**Figure 2:**
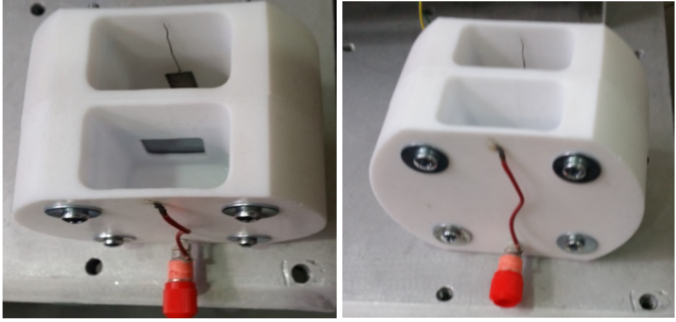
Anodization cell designed by us.

In this work, we used double tank electrochemical cell to fabricate porous silicon, shown as Figure 2 [18]. Each sample was vertically placed in a home-made Teflon double-tank cell, which consists of 2 platinum electrodes at the same length and shape.

For 4 mg/cm^2^ Pt loading, 78.9 mg Tanaka catalyst, 225.9 mg Nafion solution, 4 mL 2-proponal, and 2 mL deionized water were mixed to obtain a catalyst solution. By brushing, the carbon papers with loading of catalyst solution were used as the gas diffusion layer (GDL). Carbon papers, which has 4.41 cm^2^ surface area, were cut and prepared at the same length and shape for anode and cathode electrodes.

The contact of the GDL with the membrane was achieved by the silver paste. Furthermore, since GDL is conductive, we did not need external flow collection plate for current collection

### 2.4. Fuel crossover

To prove that the produced porous silicon works as a membrane, 2 main measurements that are fuel crossover test and electrochemical impedance spectroscopy have performed. The fuel crossover test provides information about the formation of pore along the silicon wafer and the fuel crossover affecting the fuel cell performance of the membrane was investigated. The undesired crossover of methanol from the anode to the cathode through the membrane is important because it degrades fuel cell performance. There are 3 types of transport mechanisms that cause the methanol crossover through the membrane. These mechanisms are namely electro-osmotic drag by proton transport, diffusion by methanol concentration gradient, and convection by the hydraulic pressure gradient between the anode and the cathode. Methanol crossover refers to which the methanol passes to the cathode compartment where it gets oxidized along with oxygen reduction reaction, thus resulting in poor cell performance [19]. In addition, since the oxygen to be used for the reduction of hydrogen is consumed, the oxygen that enables the main reaction to take place decreases.

Rhee, Ha, and Masel [20] showed that formic acid was slower than methanol and formic acid could be used as an alternative fuel in proton-conducting membrane fuel cells. Therefore, formic acid was preferred as fuel in a fuel crossover test.

In this study the fuel crossover test was performed in the double-tank cell (see Figure 2). 50 mL of deionized water was placed on the cathode side of the cell and 50 mL, 5 M formic acid was placed on the anode side. Current change was observed using Keithley 2400 at room temperature without applying an electric field.

### 2.5. Electrochemical impedance spectroscopy

Electrochemical impedance spectroscopy was performed to determine the proton conductivity of the produced porous silicon. For impedance spectroscopy measurements, 1 M sulfuric acid solution was placed on the cathode side and 5 M formic acid solution was placed on the anode side of the Teflon cell. Between the 2 electrodes, a signal with an amplitude of 400 mV in the frequency range from 20 Hz to 10 kHz was applied at 25 °C and the Nyquist Z plot obtained with the measurement results using the EDC-1630 digital LCR meter for proton conductivity.

## 3. Results and discussion

### 3.1. Morphological characterization of porous silicon

**Figure 3:**
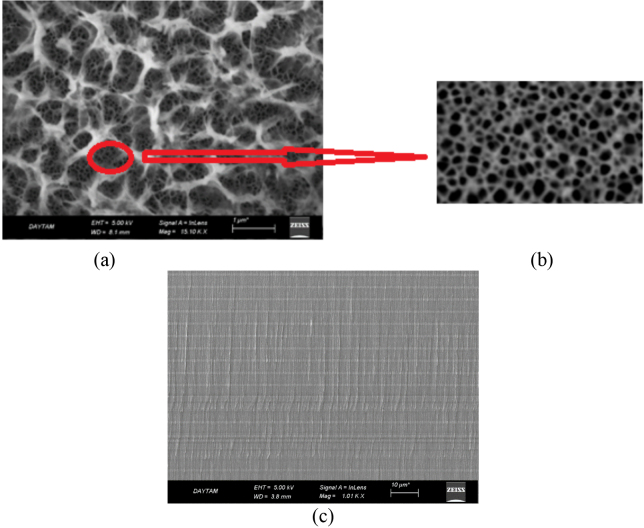
a)-b) Surface SEM images of porous silicon c) cross sectional SEM images of porous silicon.

The structural characterization of the porous silicon was analyzed by SEM. Figure 3 (a, b, c) shows SEM analysis of the layered structure which was formed by the application of current densities of 1,2,5,10 mA/cm^2^ in a specific order. Anodization time and the growth pore depth were controlled by the layered structure. The membrane produced had a porosity of 93% and pores with diameters ranging from 20 to 25 nm. Table 1 shows etching thickness as a function of current density.

**Table 1:** Etching thickness as a function of current density at J = 1 mA/cm^2^, 2 mA/cm^2^, 5 mA/cm^2^, and 10 mA/cm^2^.

Current density (mA/cm^2^)	Etching time (min)	Etching thickness (μm)
1	40	1
2	40	2
5	40	5
10	40	10

**Figure 4:**
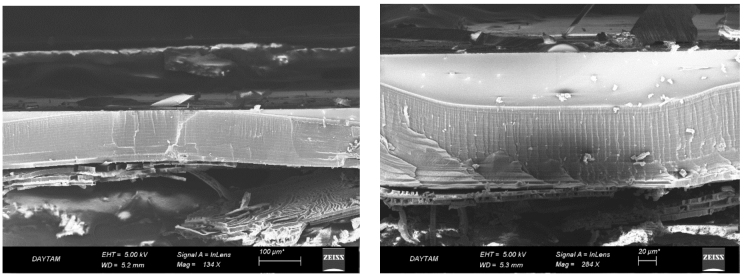
SEM image of porous silicon.

As seen in Figure 4, in the areas masked with PVC film, we observed that pore does not form until the adhesive of PVC film loses its function. This shows that the masking was successful. However, in nonmasked areas, the thickness of the porous layer was formed along the silicon wafer. SEM analysis showed that one-third of the required anodization time throughout the thickness of the silicon wafer causes the full removal of the PVC film from the surface with the ethanolic HF solution. On the other hand, if the thickness of the silicon wafer to be used as a membrane is adjusted, it is possible to completely prevent the formation of pores in the masked areas of the silicon wafer.

### 3.2. Fuel crossover test

**Figure 5:**
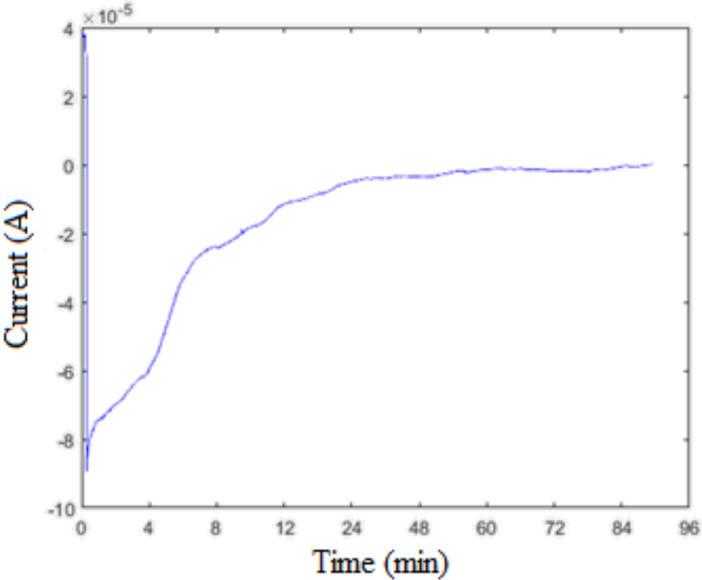
*I − t* change of porous silicon membrane for 5 M of formic acid transition.

Figure 5 shows the obtained current in the fuel crossover test. However, after about 60 min, the amount of change of the current was considerably reduced, had been fixed over time, and reached zero value. Ion transfer was performed during the period of change of current. The moment that the current is fixed and zero indicated that the concentrations are equalized and the ion passage is complete. To observe the pH change due to the fuel passage, pH values were measured at the side of deionized water every 15 min.

**Figure 6:**
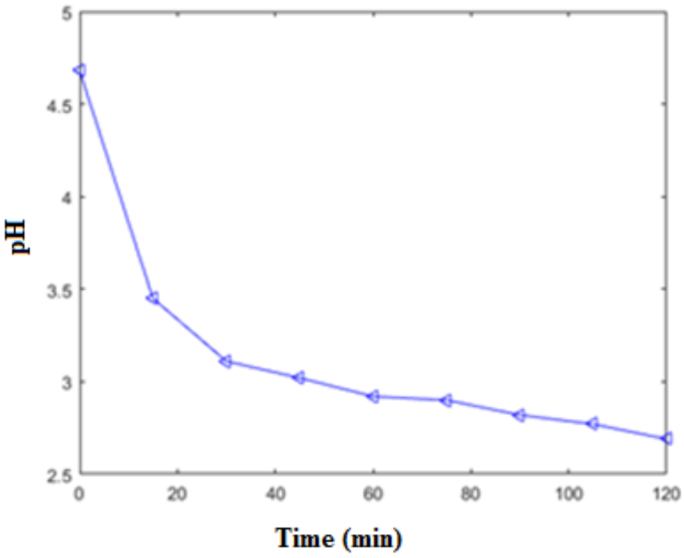
Time-dependent change of pH for the formic acid transition.

pH measurements were made using Mettler Toledo SevenCompact pH meter. As seen in Figure 6, the pH values changed from neutral to acidic values, and the ion exchange was reduced after 60 min. In light of these results, it can be said that the pores were opened along the silicon wafer and the permeability of the membrane was examined. The results of the measurements shown in Figure 5 and Figure 6 seem consistent. Both results also showed that the fuel cell can provide energy for about 60 min with a 50 mL fuel.

### 3.3. Proton conductivity

**Figure 7:**
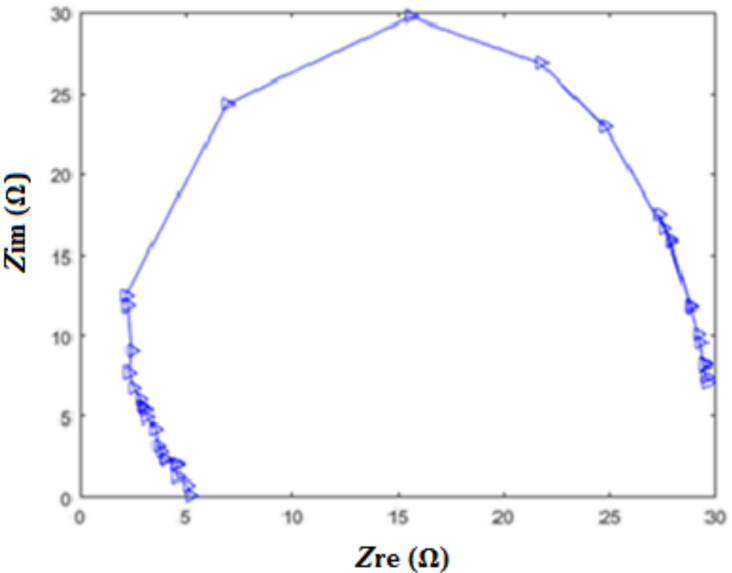
The Nyquist Z plot of porous silicon membrane.

A semicircular structure was observed in Nyquist Z plot that is shown in Figure 7. This symbolizes a parallel circuit. Table 2 shows Fhe results obtained from electrochemical impedance spectroscopy (EIS) test.

**Table 2:** Proton conductivity for nanoporous silicon membrane.

S(cm^2^)	R_m_(Ω)	σ (S/cm)
2.56	5	0.0016

Proton conductivity, which is detected by the electrochemical impedance spectroscopy is used to determine the amount of ion transported [9]. The proton resistance per unit area can be obtained using Nyquist Z plot. The proton conductivity of the membrane was calculated using Eq. 1 [17];

(1)σ=dRmxS

The conductivity of the membrane was calculated as 0.0016 S/cm by using Eq. 1. In Eq. 1, *d* represents the thickness of the membrane and *S* is the cross-sectional Pt contact area.

### 3.4. Fuel cell performance

**Figure 8:**
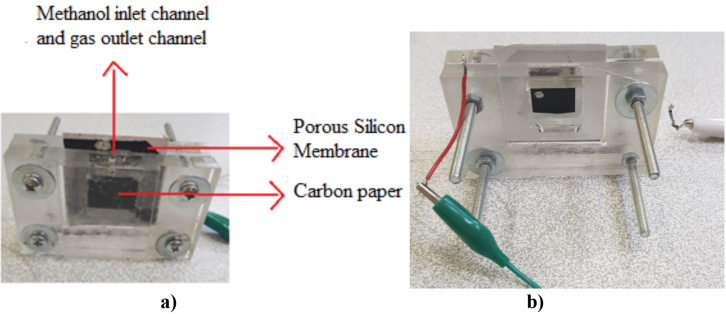
μDMFC prototype cell a) anode b) cathode.

We measured the performance of fuel cell at room temperature. A constant voltage was applied that ranged from 0.9 V to 0.1 V in steps of 0.05 V using Keithley 2400. Figure 8 (a, b) shows the prototype fuel cell.

Due to the electrical conductivity of carbon paper, we did not use an additional element to collect current so that the structure of the cell has become simpler. In this way, the use of graphite layers and gold was eliminated.

**Figure 9:**
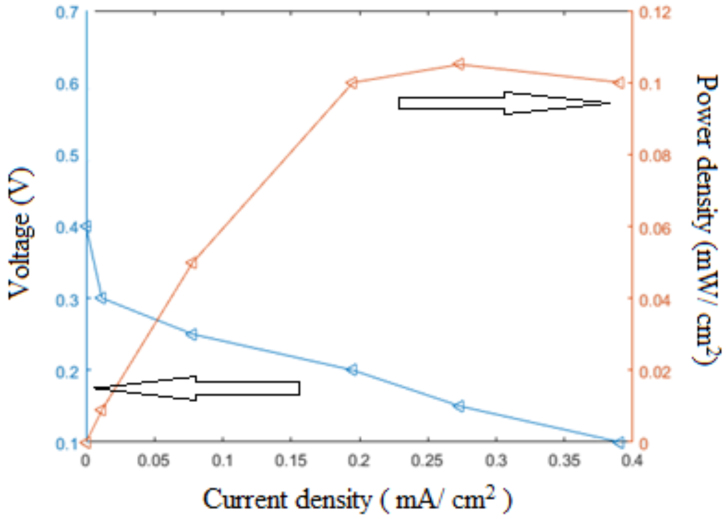
*I − V* and power density characteristic of the miniature fuel cell made with a PSM.

As seen from the fuel cell performance measurements given in Figure 9, the open circuit voltage (OCV) of the system is 0.4 V, the highest power density is 0.1 mW/cm^2^, and the highest current density is 0.39 mA/cm^2^. The OCV value is one of the parameters that shows fuel cell performance. In the case of proton conducting fuel cells using Nafion as membrane, the OCV value is about 0.9. However, porous silicon is not naturally protonconducting, and it provides proton conduction through only nanopores. Previous studies [12,14,15,17] noted the OCV values in a range between 0.3V and 0.7V. The acidic property is a parameter that enhances proton delivery. Although previous suggested [14,16,17] that acid deposited within the pores may increase the performance of fuel cell, we did not apply it due to the focus of this study on the porous silicon membrane production method, rather than the performance of the fuel cell. Table 3 presents a comparison of the results between our method and another study in terms of conductivity.

**Table 3:** Comparison of the proton resistance per unit area of the PEMs based porous silicon at 25 °C.

Membrane	d(cm)	R_m_ (Ω)	σ (S/cm)
This paper	0.2	5	16*10^-4^
[16]	0.01	288.59	1.77*10^-4^

## 4. Conclusions

This study paves a new way for a more cost-effective, and short time-consuming method for porous silicon, and the findings of the analysis represent the first experimental evidence of the aforementioned production method. In this respect, EIS, fuel crossover test, and fuel cell performance tests were performed at room temperature to examine the performance of the porous silicon produced in passive mode μDMFCs as the proton-conducting membrane. On the other hand, there are a variety of factors influencing on the performance of μDMFC, such as methanol concentration, methanol flowrate, operating temperature, humidity of reactants, and types of catalyst. Following the production method that was presented in this study, future studies may consider optimizing other parameters to increase the performance of μDMFC.
